# DNA of centuries-old timber can reveal its origin

**DOI:** 10.1038/s41598-020-77387-2

**Published:** 2020-11-23

**Authors:** Linar Akhmetzyanov, Paul Copini, Ute Sass-Klaassen, Hilke Schroeder, G. Arjen de Groot, Ivo Laros, Aoife Daly

**Affiliations:** 1 Chair of Forest Ecology and Forest Management, Wageningen University and Research, Wageningen, The Netherlands; 2 Wageningen Environmental Research, Wageningen University and Research, Wageningen, The Netherlands; 3 Thünen-Institute of Forest Genetics, Grosshansdorf, Germany; 4 The Saxo Institute, University of Copenhagen, Copenhagen, Denmark

**Keywords:** Forest ecology, Forestry

## Abstract

Oak wood was highly appreciated and widely used for construction in past centuries. As population sizes expanded in some regions of Europe, local forests were depleted of high-quality timber. Therefore, regions of soaring economies were importing timber initially from the European market and eventually from other continents. Origin of archaeological or historical timber is usually identified by means of dendroprovenancing, i.e. statistical matching of tree-ring-width (TRW) series of timber of unknown origin with TRW reference datasets. However, this method has pitfalls and limitations and therefore alternative techniques are needed. Here, we used three different DNA analysis methods to investigate the potential of using ancient (a)DNA, extracted from oak timber derived from historical buildings and shipwrecks from a variety of countries. All the material had also been analysed dendrochronologically, so its dating and provenance is demonstrated. We included heartwood samples in this analysis, for which DNA extraction is especially challenging as it contains chemicals that inhibit DNA amplification. We succeeded in amplifying DNA for at least one marker from 56% of samples (including heartwood samples), yielding crucial information that allowed us to identify the potential source area of centuries old timber buildings in Latvia and Denmark and of 750-year-old shipwreck material from Germany. Our results prove the strong potential of DNA analyses for identifying timber origin to the regional scale, but by combining these with the dendrochronological results, we can control the exactitude of the aDNA approach and demonstrate a more nuanced examination of the timber sources for these historic structures.

## Introduction

Demographic evolution during the Middle Ages in Northern Europe and during the Age of Discovery in the Iberian peninsula^1^ resulted in an increased need for construction timber which in turn had an impact on forest cover in these areas^2^. Particularly around the growing market towns, local forests could hardly sustain the increased demand in primarily high-quality timber for shipbuilding, construction of monumental buildings, such as cathedrals^3^ and urban infrastructure^4^. Vast amounts of wood were imported and transported from elsewhere, mainly by rafting via the rivers or by shipping^5,6^ even from other continents^1^.

Oak wood (*Quercus* spp.) was highly appreciated for its mechanical properties and its durable heartwood^7^, and oak timbers of widely varying dimensions are commonly found as archaeological remains e.g.^8,9^ and in standing historic buildings. These remains are valuable archives for retrieving the age and the origin of timber which in turn provides information on local availability of timber resources and the organization of past timber trade e.g.^10–13^. Until now, the dendroprovenancing method, i.e. matching tree-ring width (TRW) series of timbers with a geographical network of reference TRW chronologies existing for the species^14^ has been the most common approach to suggest the source area of timber e.g.^15–17^. However, this method has some limitations, for instance, timbers with a limited number of tree rings, as sometimes found in historical timber e.g.^18^ often cannot be dated and thus their source cannot be identified. Additionally, some timbers lack a strong climatic signal in TRW series, limiting precise identification of timber source areas^19^. Tests on using other tree-ring related wood-anatomical^20^ or wood chemical^21^ time series as well as wood-chemical profiles^22^ revealed the power and weakness of each method, but also the potential of integrating different approaches^20^. Adding DNA analyses to the toolbox may further enhance the success of provenancing efforts. First trials for oak-timber provenancing indicated that such methods hold a large potential, but need to be improved^23,24^.

The genetics of ring-porous European oaks has been intensively studied, and altogether 32 distinct haplotypes were defined, from trees growing across Europe^25^. The availability of such an extensive dataset on oak haplotype distribution potentially allows pinpointing wood origins from regional up to country level within the continent, as a strong phylogeographic structure was observed among these haplotypes. Some of the haplotypes are spread over large areas but some are unique for certain regions and thus can be used for identification of timber origin. Moreover, Schroeder et al.^26^ developed a set of DNA markers for the identification of oak timber origin on a continental level. These markers can be used as a first step to identify the continental source (i.e. Europe, Asia or North America) of archaeological oak timber. Thus, using markers first for continental provenance identification and then using chloroplast single sequence repeat (cpSSRs) markers (microsatellites) developed explicitly for haplotype identification of white European oaks^27^ would, in theory, allow pinpointing potential source areas.

Although reference datasets are to some extent available, application of DNA-based provenancing methods has so far been hampered by the quality of DNA that can be extracted from old and partially degraded wood samples. DNA extraction from historical timber is challenging, as old timber has been exposed to abiotic (UV radiation, changes in moisture content resulting in shrinkage and swelling) and biotic (microorganisms) factors that mechanically disrupt cells or degrade DNA fragments by exogenous nucleases^28^. While extracting the required amounts of DNA of sufficient quality is already difficult for historic sapwood, this is even more complicated for the heartwood (the inner part of the tree). Sapwood contains living (parenchyma) cells at the time when the tree was cut, whereas all heartwood cells, even in the living tree, are already dead for many years^29^, meaning that the DNA is partly degraded even before the tree is felled. Moreover, heartwood contains extractives, which increase the resistance of cell walls to degradation but may inhibit DNA amplification^30,31^. The few studies done on DNA extraction from the heartwood of archaeological timber showed limited success expressed in low amplification rates^23,32,33^ whereas a recent study based on the sapwood of the same type of timber showed promising results^34^. However, finding a sufficient amount of sapwood preserved in historical or even archaeological timber is a challenge, as often either only the more durable heartwood was used for construction purposes or sapwood that remained attached has decomposed.

### Sample selection

In this study, we test two DNA extraction protocols and analysis methods on ancient oak timber from dendro-dated historical structures in Spain, Denmark, and Latvia as well as from archaeological ship timber to identify their geographic origin. All site locations are listed in Table 1. A total of 13 sapwood and 17 heartwood samples (*Quercus* spp*.*) were collected from 17 historical buildings. The number of timbers ranged from five in Northern Spain and Denmark to seven in Latvia. In order to test the possibility of DNA extraction from archaeological timber, considered as the most complicated case due to all the degradation processes, material from four shipwrecks (two sapwood and nine heartwood samples) was collected. While the samples from the historical buildings are untreated, the samples from the archaeological structures have very varied preservation conditions and conservation treatments after they were excavated. One major strategy in selecting samples was to examine the potential of the method in samples with varying preservation history. Timbers from standing buildings might have remained very stable after the tree was felled, so that potentially their DNA would be less decayed, and in a stable state. Of the timbers from Spain, three are dating to the eighteenth century and two are from mid-sixteenth century. All of them were collected from historic buildings from Northern Spain. Three of the samples are roof beams from the San Martin de Tours Church and Asuncìon church, whereas the remaining two were collected from the staircase of the church in San Vicente de Arana. The timbers from Denmark are from two historic buildings. Two are from beams in a timber-framed townhouse in Aalborg in North Jutland. They are both from trees felled in c. AD 1560s–1570s and the dendrochronological analysis of these samples suggest that these trees had grown on the Swedish side of Kattegat (which in the sixteenth century was still Danish territory). The other three samples from Denmark are from beams in a timber framed house in Horsens, on the east of the Jutland peninsula. The three probably represent two felling phases, one c. 1614–1622 and another 1621–1622. In contrast to the timber from Aalborg, the timber from Horsens is probably of local origin. The timbers sampled from Latvia are from two separate buildings. Two beams and a plank from a door are from St Jakob’s Church in Riga. The plank from the door is dating to late fifteenth century, one beam is from around the early decades of the sixteenth century while the other is from late seventeenth century. The trees that these were made from might come from the Riga hinterland or inland from Riga, transported perhaps by the Daugava River. Dendrochronologically however this is still an open question. The Riga material is dating with so-called art-historical chronologies^35^, based on oaks used as supports for artworks, that was shipped to Western Europe from the east and south Baltic region over many centuries. The second Latvian building is the castle at Cēsis. The early sixteenth century arquebus is also dating with the art-historical chronologies, and might be from the castle hinterland.

**Table 1:** Overview of the samples collection and the DNA results per study object.

Object code	Location	Object	Material	Dated to	Wood type	CO	HP	Collector
JMT011	Spain	Staircase	H	1777	SW/HW	Eu	Not 7	MDD
JMT031	Spain	Staircase	H	1779	SW/HW	Eu	Not 7	MDD
ARR031	Spain	Roof beam	H	1762	SW/HW	NA	1	MDD
SVT031	Spain	Roof beam	H	1548	SW/HW	NA	Not 7	MDD
SVT061	Spain	Roof beam	H	1548	SW/HW	Eu/As	10, 11	MDD
H011001	Denmark	Beam	H	1564–1579	SW/HW	Eu/As	10, 11	AD
H011005	Denmark	Beam	H	1560–1575	HW	NA	NA	AD
F042003	Denmark	Beam	H	1614–1622	SW/HW	Eu/As	1	AD
F042010	Denmark	Beam	H	1608–1623	SW/HW	Eu	1,7	AD
F042011	Denmark	Beam	H	1621–1622	SW/HW	Eu/As	1,7 not 7	AD
JK-I	Latvia	Beam	H	~ 1514(= 1493)	HW	Eu	10, 11, 12	MZ
JK-B	Latvia	Beam	H	1678(?)	HW	NA	7, 10, 11, 12	MZ
JKd2	Latvia	Door	H	~ 1479(= 1477)	SW/HW	Eu/N Am	12	MZ
CesDt2	Latvia	Arquebus	H	~ 1538	SW/HW	Eu/As	1, 7	MZ
CesDt3	Latvia	Arquebus	H	~ 1538	SW/HW	Eu/As	1, 7	MZ
CesDt5	Latvia	Arquebus	H	~ 1538	HW	NA	NA	MZ
CRL32	Latvia	Beam	H	~ 1278	SW/HW	NA	NA	MZ
Z255002	Germany	Shipwreck	A	~ 1291–1307	SW/HW	Eu	12	AD
Z018.605.029	USA	Shipwreck	A	After 1476–1505	HW	NA	NA	AD
Z018.605.040	USA	Shipwreck	A	1546–1591	HW	NA	NA	AD
Z0922319a	Sweden	Shipwreck	A	1490–1581	HW	NA	NA	AD
Z092332a	Sweden	Shipwreck	A	1479–1612	HW	NA	NA	AD
Z092313a	Sweden	Shipwreck	A	1381–1620	HW	NA	NA	AD
Z092314a	Sweden	Shipwreck	A	1434–1621	HW	NA	NA	AD
Z092324b	Sweden	Shipwreck	A	1445–1569	HW	Na	NA	AD
Z223004	Germany	Shipwreck	A	1419	SW/HW	Eu/As	10	AD

*Dated to* an estimated cutting date based on sapwood statistics or radiocarbon dating; *H and M* historical and archaeological material type, respectively; *SW and HW* sapwood and heartwood, respectively; *CO* continental origin identified; *HP* haplotype identified; *As, Eu, N Am and NA* Asia, Europe, North America and “not available”, respectively; *MDD, AD and MZ* M. Domínguez-Delmás, A. Daly and M. Zunde, respectively.

The archaeological samples are from four shipwrecks. Two timbers from the so-called Sparrow Hawk, a wreck exposed and lifted in the 1860s from Cape Cod in Massachusetts, USA were included. This wreck has been hailed as the ship described by William Bradford^36^ that carried English and Irish to the New World in 1626^37,38^. This material is dated using C14 analysis, to the late sixteenth century^39^, and the DNA analysis might show whether the wood was of European or American origin, allowing us to come closer to suggesting whether the origin of this ship fits the legend. The timbers seem to be untreated, but they have dried out. Timbers from the warship *Vasa* which sank in Stockholm harbour in 1628^40,41^ were also included for analysis here. This ship in its entirety was lifted out of the harbour in 1959 and underwent conservation with Polyethylene glycol (PEG). It now stands in a purpose-built museum in Stockholm and current dendrochronological analysis of its timber is demonstrating several timber sources for the building of the ship, including eastern Swedish and east Baltic sources^42^. A timber from a shipwreck found off the coast of Mecklenburg-Vorpommern (Peenemündung, Ostsee VII, FPL105^43^) was also selected for aDNA analysis. The dendrochronological analysis shows that the ship was built c. 1419–1420 and the sub-sampled plank for this study suggests a southern Baltic source for the timber^44^. The excavation took place in January–February 2018 and dendrochronological analysis of this sample was completed by April that same year. However, after analysis the dendrochronology sample, from which the sub-samples for aDNA were extracted, were allowed to dry out. Finally, a sample from another wreck from the coast of Mecklenburg-Vorpommern (Mönchgut, Ostsee VII, FPL100^45^) was sub-sampled for this study. The wreck was found in 2014 and re-inspected in 2018. The dendrochronological dating shows that the wreck is from c. AD 1272–1307 and the timbers seem to have grown in southern Sweden^46^. The dendrochronology samples and the sub-samples for aDNA analysis were kept wet, in sealed plastic containers.

In total 41 samples (15 sapwood and 26 heartwood) from 26 objects were analysed. They represent historical timber of a range of dating and preservation and conservation conditions allowing a thorough test of the methods used for DNA extraction and detection. All these samples were previously dendrochronologically or radiocarbon dated, and ages ranged from the thirteenth to seventeenth century (Table 1). Based on the anatomy all objects were identified as deciduous oak species (*Quercus* spp.).

We also included sapwood and heartwood from contemporary trees as a reference (respectively five *Quercus petraea, Q. faginea* and *Q. pyrenaica,* and three *Q. robur* trees). All contemporary oaks are growing in forests located in the Basque Country, Northern Spain (42° 43′–42° 56′ N; 2° 48′–2° 51′ W) where trees were intensively logged in the past for ship construction. In October 2016, one wooden sample was taken from each tree by using a 40 cm long Haglöf increment borer. Obtained samples were put into zipped plastic bags with silica gel.

The samples from historical and archaeological objects were sent to the Molecular Ecology Laboratory of Wageningen Environmental Research, Wageningen, The Netherlands for DNA analyses. Historical samples were also analysed in the Thünen Institute of Forest Genetics, Großhansdorf, Germany. Historical, archaeological and fresh samples were always treated completely separated, i.e. kept and analysed in different lab rooms to avoid contamination.

Extraction protocols were tested in two independent laboratories on subset material from the same samples. The details of the methodology are described in the materials and methodology section of this paper. In the results presented here we highlight three case studies to illustrate the added value of integrating DNA into provenance studies and the relevance for applied archaeological investigations. We hypothesize that DNA can be extracted from sapwood and, albeit in smaller quantity and quality, also from heartwood. This DNA can then be used for provenance identification of oak timber (a) on the continental level and (b) on regional/country level.

## Results

### Comparison of success of methods

**Figure 1:**
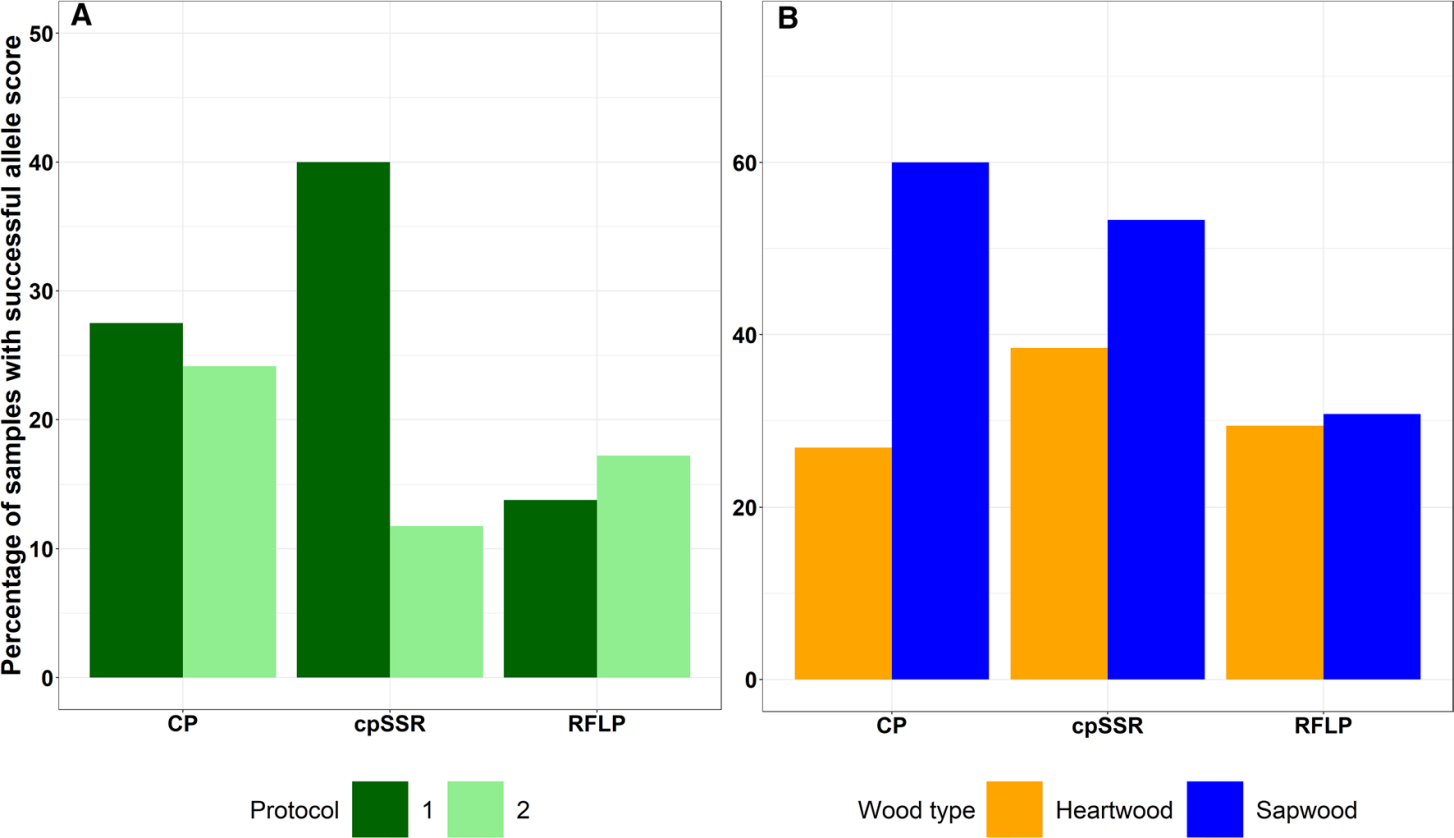
Percentages of samples with successful allele scores per extraction protocol (**a**) and per wood type (**b**). CP, cpSSR and RFLP refer to continental provenance, microsatellites and restricted length polymorphism analyses, respectively. Samples with at least one marker scored per provenance identification method are considered as successful.

For 56% of the total amount of oak samples (23 historical or archaeological samples, 13 were heartwood and 10 sapwood with ages varying between 241 and 729 years) allele scores (fragment sizes) were obtained for at least one of the provenancing methods tested, although rarely scores were obtained for all markers of a method (Table S1). Success percentages differed between combinations of extraction and provenance determination methods (Fig. 1a). Both the highest and lowest success percentages were observed for the microsatellites based haplotyping method, depending on the extraction protocol used. For all provenance determination methods per type of wood more sapwood than heartwood samples showed successful allele scores, though for the RFLP analyses, results between the wood types were similar (Fig. 1b). Of a total of 26 heartwood samples, allele scores were obtained at least for one marker from 13 samples (50%, Table S1), with a higher success rate for historical samples compared to archaeological samples (64.7% and 22.2%, respectively). Wherever multiple results for the same object were obtained (e.g. from two extraction methods or both for heartwood and sapwood), the results always confirm each other (as can be seen in Table S1) and cross validated in two independent laboratories. No significant difference in amplification success rates was observed between the two dilutions (data not shown).

#### The big picture—continental provenancing

Using the contemporary trees (using both heartwood and sapwood), we found the same alleles and their combination in *Q. pyrenaica* and *Q. faginea* from Spain and other European oak species, such as *Q. robur* and *Q. petraea* (Table S3). In total, for 77% of samples, DNA was successfully amplified and fragment sizes could be scored. From this subset, all samples showed scores indicating a European origin.

Among the historical and archaeological set of samples with at least one allele scored, samples from five objects were identified as “European oak” (Table 1) and 11 samples were identified as having either Eurasian or European/North American origin (Table S3). Out of these 16 samples, nine were sapwood and seven of heartwood type, comprising 26.9% and 60% of the total number of respected wood type samples, respectively (Fig. 1b).

A similar success percentage between extraction protocols was achieved for the continental provenance determination analyses: 27.5% and 24.1% using Protocol 1 and Protocol 2, respectively (Fig. 1a).

We already know that the majority of the archaeological and historical material in this study is of European origin through the dendrochronology, so these results are not in conflict with the knowledge we already had. For the one wreck where this detail would have been a very useful result—the Sparrow Hawk from Cape Cod—unfortunately no alleles were scored.

#### Regional provenance analysis by haplotype determination

For the microsatellite analyses, amplification percentage strongly depended on the DNA extraction method used, with Protocol 1 showing higher success than Protocol 2 (40% versus 11.7% respectively, Fig. 1a and Table S1). At the same time, RFLP analyses were more successful for DNA extracted via Protocol 2 (17.2%, versus 13.8% for Protocol 1, Fig. 1a and Table S1).

Based on the microsatellite analyses, at least one allele was scored in ten heartwood and eight sapwood samples (38.5% and 53.3% for heartwood and sapwood, respectively, Table S1). The exact haplotype of seven out of 26 objects was identified (27%), while for nine objects at least one allele score was obtained at least for one marker (Table S1). However, while results for multiple markers together make up one haplotype, some of the haplotypes can already be recognized based on one or a few unique alleles at some markers. For example, for the object ARR031, two markers were enough to identify its haplotype and for the object F042011, results from combined RFLP and cpSSR methods allowed to identify its haplotype (Table 1 and Table S1). With the RFLP analyses, at least one allele of nine samples [five heartwood (29.4%) and four sapwood (30.8%)] were scored for at least one marker (Fig. 1b), and two samples were identified as having haplotype 1 and haplotype 7. It is clear that results were achieved primarily with the samples from historical buildings. Especially the dried out archaeological wood produced meagre results. It is noteworthy however, that for two archaeological samples (from one timber (sapwood and heartwood) from shipwreck Mönchgut (Ostsee VII, FPL100^45^, Z255002), which are considered as the most complicated wood material due to the degradation level of the samples, alleles were scored for almost all the used markers. This particular dendrochronological sample and the subsequent sub-samples for DNA analysis had been kept waterlogged since excavation.

### Case studies—old, older, oldest

As mentioned above, three case studies are highlighted from this experiment, as they provided useful results demonstrating added value of aDNA for timber provenancing.

#### 400-year old historic house in Horsens, Denmark

Three samples were collected from a seventeenth century historical building located at Nørregade 12, Horsens, Denmark (samples F042003, F042010, and F042011 in Table 1). The houses in this main street of the town are of great architectural and historical value^47^. According to dendrochronological dating results, the samples are dated to 1614–1622, 1608–1623, and 1621–1622, respectively, and probably came from trees that grew in the hinterland of the town^48^. However, the three timbers dendrochronologically belong to two separate provenance groups. Haplotype analyses revealed that sample F042003 (heartwood, based on the CpSSR analyses) belongs to haplotype 1, whereas both cpSSR and RFLP analyses showed that sample F042010 belongs to haplotype 7 (Table S1).

**Figure 2:**
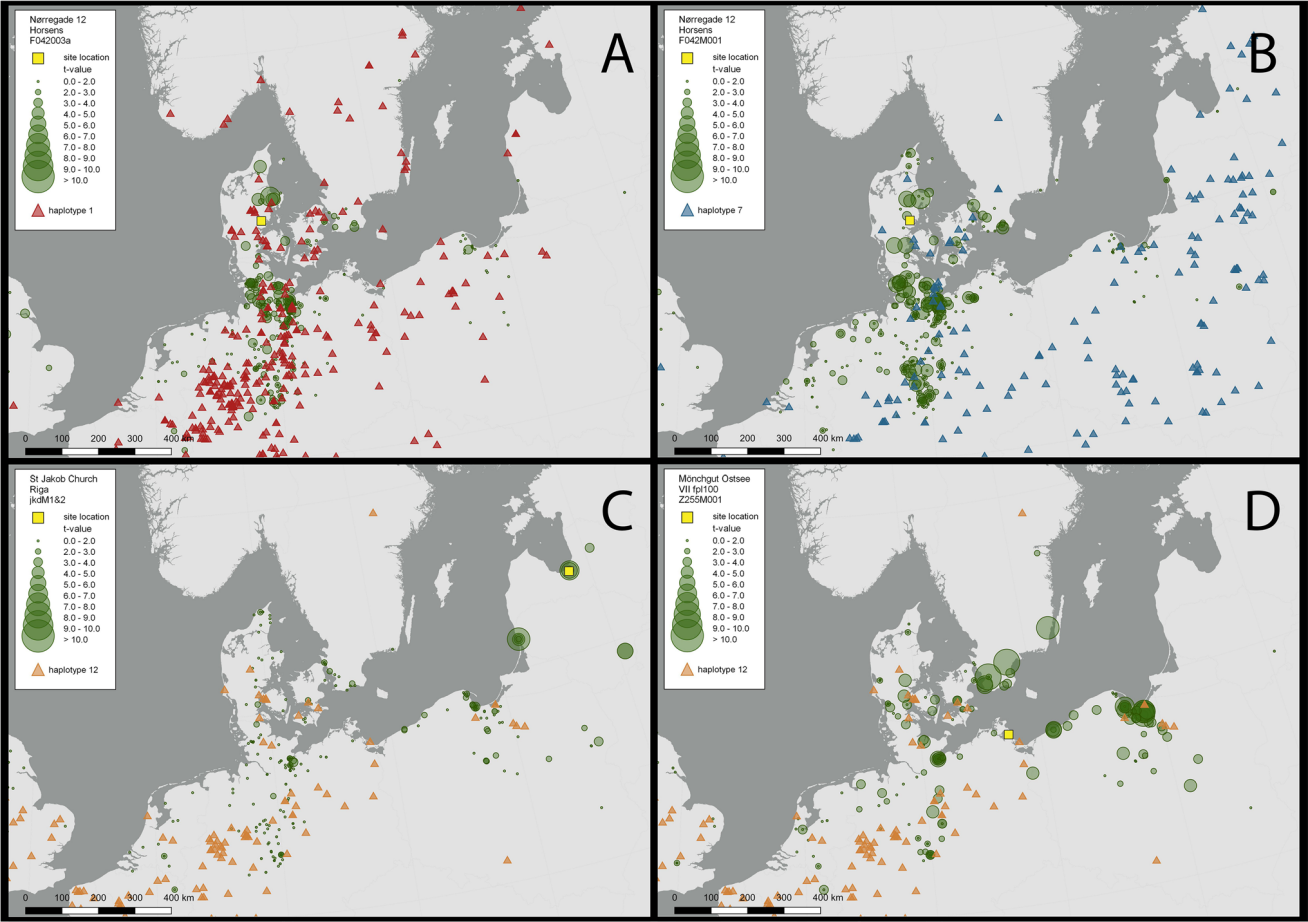
Potential timber source area of the historical house in Horsens (**A**,**B**), Riga church door (**C**) and Mönchgut Ostsee shipwreck (**D**). The object location is shown (yellow square). The *t*-values are indicated by the size of the circle and the highest values are given (for a detailed description of the technique see e.g. Daly^17^). The haplotype distributions (after Petit et al.^49^) are indicated by triangles (see maps legends). Map is created using QGIS Desktop 3.4.12 (https://www.qgis.org).

Both haplotypes identified (HP1 and HP7) are matching the haplotype distribution that would be expected for Denmark. Haplotype HP7 has a Balkan refugial origin and has a wide distribution over Europe reaching the Baltic States^49^. As for Denmark, HP7 is mainly found in the eastern part of the country, whereas HP1 is the dominant haplotype in the region, comprising 55% of the trees^50^. The DNA results support the dendrochronological, in that the separate correlation groups are also genetically separate groups. In terms of identifying the provenance of these timbers, the dendrochronology provides us with a convincing indication of the source area of the trees, whereas the DNA in these cases serves rather to confirm this (Fig. 2A,B), as both haplotypes have a very wide distribution across the European continent, from southern Scandinavia to Italy^49^.

#### 650-year old church door from Riga, Latvia

The sample JKd2 (Table 1) consists of sapwood from an old door of the Saint Jacob church (or St. James’s Cathedral, or the Cathedral Basilica of St. James), which is in Riga’s Old town, Latvia. Its outermost preserved tree-ring was dated to 1477 by using the well-known *Baltic 1* and *Baltic 3* regional TRW chronologies but regional provenance of the samples remains unsure^51^. Current research is indicating that the Baltic 1 and Baltic 3 chronologies might have their source in the regions east of the Baltic Sea coast, rather than south, as has previously been assumed (Daly & Tyers, in prep). Based on the cpSSR analyses, the samples were identified as belonging to haplotype 12 (Fig. 2C). This haplotype belongs to lineage B^25^ and is one of the most common haplotypes of this lineage (see Petit*, *et al*.*^49^ for detailed information on haplotypes distribution). In modern trees, HP12 has a western European distribution with its easternmost limit represented in outlying populations around Gdansk^49^. We might reconcile the somewhat conflicting indications between the dendrochronology and the DNA analysis by suggesting that the HP12 group might have had other outlier groups of population further east in the past? Huge exploitation of oaks over centuries in this region might mean that these outliers did not survive into the modern populations.

#### A 700-year-old shipwreck from Northern Germany

From the collection of archaeological material, alleles were scored for almost all used markers for both heartwood and sapwood of the sample Z255002 (Table S1) demonstrating the necessity to keep waterlogged archaeological samples from drying out to maximize preservation of ancient DNA.

The sample was collected from the Mönchgut Ostsee shipwreck off Rügen, Northeast Germany. It was dendrochronologically dated to 1291–1307 and had the highest correlations with South Swedish chronologies^46^. Continental provenancing analyses of the sample confirmed its European origin and based on cpSSR analyses the sample was identified as belonging to haplotype 12 (Table 1), the same haplotype as the medieval door from Riga. This predominantly western European haplotype is one of the most common haplotypes in Central Europe in the modern tree study and is widely distributed across Northern Germany, and reaching forests in Southern Denmark^52^ but for the rest of Scandinavia it was only found in some trees from Central Sweden^50^. Again, there is a possible discrepancy between the dendrochronology and the DNA, when we compare the ancient DNA with modern-day oak populations.

## Discussion

### DNA from wood for timber provenance identification.

#### A new player

Current dendroprovenance methods based on TRW, isotopes, mass spectrometry or wood anatomy were successfully applied in many studies e.g.^15,22,53^. However, each method has its own limitations and specific requirements for successful identification of timber origin, e.g. a sufficient number of rings, specific climatic gradients in the study area or distinct differences in bedrock material etc. Additional methods such as DNA-based source tracking is therefore relevant to enhance the precision of timber provenance identification as it contains alternative information on timber origin which is independent of growing conditions and also can be used on a small amount of material. However, such methods require improvement of DNA extraction and amplification protocols, especially when dealing with old and partly degraded material. Particularly, until now, DNA extraction from waterlogged archaeological timber had shown limited success^23,54^. In our study, we demonstrated the possibility of extracting DNA from timber at different degradation states by amplifying DNA and reconstructing the exact haplotype of seven timbers and identifying the continental origin of four objects out of 26. Using different DNA analysis methods provided complementary results, e.g. sample F042011 from Denmark, indicating the importance of using multiple techniques for achieving the best results.

#### A strong combination: ancient DNA and dendrochronology

We could show that in 56% of all samples, allele scores were obtained for at least one marker per provenance identification method, both from sapwood and heartwood. However, our DNA results look still incomplete because in most cases only some markers for some combinations of DNA-based provenance identification methods could be successfully amplified (Table S1). Nevertheless, we have demonstrated the potential of using ancient DNA from oak for identification of timber origin. Even limited information that is sometimes retrieved from the DNA analyses of ancient timber is helpful for identifying timber provenance, as it can exclude certain haplotypes, e.g. QT7 marker in the RFLP analyses for haplotype 7, and, consequently, exclude areas where such a haplotype is found to be distributed. The results of the case studies also illustrate the added value of combining DNA analyses with classical TRW based dendroprovenance, as some haplotypes have a wide distribution but in combinations with the dendrochronology we can achieve a finer-scale timber provenance determination. On the other hand, the origin of the material from the church door from Riga is not yet identified based on the TRW analyses alone, whereas DNA might provide us with initial ideas on the timber source area based on the distribution of the identified haplotype and the TRW chronologies used to date the object.

#### Waterlogged archaeological timber—the most challenging

Wooden archaeological material is not always suitable for the tree-ring analysis, and some timber that is suitable for analysis can nevertheless not be dated, and therefore the origin of the material cannot be determined. This is particularly an issue in the study of shipwrecks, especially remains from the modern era, when travel reached all corners of the globe. Wood from the New World can have been used for repairing European ships while abroad. For example, American oak timber import for shipbuilding to the Spanish Kingdom has also increased in the Age of Discovery^5^. Therefore, the possibility of finding non-European timber in ‘European’ shipwrecks should not be ruled out. Since American white oak species are anatomically very similar to European oaks, i.e. ring-porous wood with wide multiseriate axial rays, it is not feasible to identify the continental origin of archaeological oak ship timber based on its wood structure^55^. Continental provenance identification of such timber based on the DNA analyses and using previously developed markers^26^ specifically for this purpose can overcome this problem. From nine ship samples, material collected from Mönchgut Ostsee shipwreck in Rügen, Northeast Germany, showed a clear signal of European origin. Moreover, based on the combination of the identified haplotype with the dendrochronological results, its potential origin is likely located in southern Sweden (Fig. 2D). Important to note that material from this shipwreck was freshly collected, i.e. was not exposed to air after archaeological excavations and that might be one of the reasons for successful DNA extraction and amplification. Certainly, for this study, the success rate for detection of aDNA in the archaeological samples was very poor, and this directly coincides with the preservation conditions and post-excavation treatment of the materials from the sites studied.

### DNA amplification from heartwood is possible

Heartwood forms a challenge for molecular studies as even in the living tree all the cells in the heartwood are dead^29,56^, and only short fragments of DNA may have remained absorbed in the cell walls^57^. In our study we confirmed that DNA can in fact be gained from ancient heartwood. DNA from 13 heartwood samples was successfully amplified (50%) and the haplotype of four of them was identified (Table S1). In previous studies, DNA from the heartwood was also amplified, but with considerably lower success rates^58^. Rachmayanti et al.^59^ have also found a gradual decrease in successful amplification rate along the wood types from outer sapwood to inner heartwood, though together with the decrease of the content of PCR inhibiting substances.

## Conclusions and outlook

Here, we show that successful DNA extraction and amplification even from old, degraded heartwood is possible and can be used for timber provenance studies. Both extraction and amplification phases show room for further methodological improvement. Our results also clearly illustrate that the integration of DNA-based and classical provenance identification methods yields new insights in historical and archaeological research. The preservation of the archaeological timber plays an important role and has to be taken into account in further studies. The logical next step is testing additional innovative DNA-based techniques, e.g. shotgun sequencing, with emphasis on shorter DNA fragments. Yet, the combination of wood genetics with more traditional dendroprovenance approaches may already further enhance the precision with which a potential origin area can be pinpointed.

## Materials and methods

### DNA isolation

The samples were first treated with UV light in the PCR cabinets for two hours per side. Then the outer layer of the sample was removed. The remaining sample was cut into pieces and 300 mg was incubated in liquid nitrogen for 2 min with a stainless steel bead (5 mm; Qiagen, The Netherlands). The frozen sample was ground using a mixer-mill apparatus Type MM 300 (Westburg) for 2 min at 20 Hz frequency. For the contemporary trees, we used 100 mg of each sample without UV treatment.

Next, we used two different DNA extraction protocols on all samples from historical objects.

- Protocol 1: DNA was extracted using the DNeasy Plant Mini Kit (Qiagen, The Netherlands) following Rachmayanti, Leinemann, Gailing and Finkeldey^60^, adding 2.6% of polyvinylpyrrolidone (PVP), into the AP1 lysis buffer (800 µl) of the DNeasy Plant Mini Kit and treating with 80 µl 0.1 M N-Phenacylthiazolium bromide (PTB), 40 µl 20 mg/ml of proteinase K and 50 µl 1 M solution of DTT before lysis incubation. Incubation period was 16 h under 56 °C.
- Protocol 2: DNA extraction was done using a previously developed and patented protocol based on the CTAB method^61^ in combination with the innuPREP Plant DNA Kit from Analytik Jena (Germany) which was used for the last purification step.

DNA from contemporary trees and archaeological objects was extracted following Protocol 1 only due to the slightly better performance of the Protocol 1 on the historical samples and due to the limited available amount of the archaeological wood.

### Determining continental origin

To determine the continental origin, we used the chloroplast marker set developed and published by Schroeder et al.^26^. First, we used DNA from the contemporary trees to test whether continental provenancing also worked for *Q. pyrenaica* and *Q. faginea*—two species that were not included in the study of Schroeder et al.^26^ but were commonly used in constructions^10^. Next, DNA from the historical objects extracted by both extraction protocols was amplified according to Schroeder et al.^26^. Due to the small amount of total DNA extracted from the historic objects, the DNA quantity was not determined and a standard dilution of 1:10 and 1:20 (DNA:water) was used for all PCR reactions.

### Determining haplotypes

The same DNA extracts and dilutions were used for haplotype identifications of the historical objects. For the latter, again, two different methods were used: an approach based on microsatellites located on the chloroplast genome (cpSSR) and a restriction fragment length polymorphism (RFLP) approach. For the microsatellite analyses, we used a total of six markers. Five of them were previously developed by^27^—*μdt1, μcd5, μkk4, μdt4,* and *μdt3*. The sixth marker—TF^25^—was used for discrimination between haplotypes 10 and 11 (see Table S2 in the Supplementary Material for the new primers sequences). Primers were designed from sequencing the whole TF fragment of non-coding DNA, following Taberlet et al.^62^.

The used set of markers allows differentiating between haplotypes 1, 7, 10, 11, and 12, five common oak haplotypes in Europe. Amplification was done in multiplex PCR using 10 μl PCR reactions, containing 2 μl template DNA, 1.9 μl H_2_O, 5 μl Qiagen Multiplex Buffer (Qiagen Multiplex Kit), 1 μl primer mix and 0,1 μl BSA (20 mg/ml). Reactions were run according to the following protocol: 15 min at 95 °C, then 45 cycles of 15 s at 94 °C, 180 s at 53 °C, 60 s at 72 °C, and finally 30 min at 60 °C. PCR products were diluted 100 times before fragment length analyses.

For the RFLP analyses, primers developed in Thünen Institute were used (see Table 2 for details). This set of markers allows identification of the haplotypes 4/5y and 5x/6 in addition to the cpSSR method. Amplification was done using 15 μl PCR reactions, containing 5 μl template DNA, 1.5 μl AmpliTaq Gold 360 Buffer, 1.5 μl MgCl2, 0.3 μl dNTPs, 0.08 μl AmpliTaq Gold DNA polymerase (ThermoFisher Scientific, Darmstadt, Germany), 0.3 μl 360 GC Enhancer, and 0.15 μl of each primer per marker. PCRs were run according to the protocol: 94 °C for 10 min, followed by 45 cycles of 94 °C for 45 s, 52 °C (54 °C for QT8F_QT5R) for 45 s and 72 °C for 45 s, and final elongation at 72 °C for 10 min.

**Table 2:** List of primers for the amplification and resequencing of the markers developed at the Thünen-Institute of Forest Genetics except for DT72^58^.

Marker name	Primer	Sequence 5′–3′	Restriction enzyme
QT8F_QT5R	QT8F	ATT TCT TGC GTT GCT ATT TAG TAA_VIC	MluCI
	QT5R	GTC ATT CGC CAC TAT GAG TC	
Dt72F_QT5R	Dt72F	GCG TTG CTA TTT AGT AAA TCC	MseI
	QT5R	GTC ATT CGC CAC TAT GAG TC_VIC	
QT7F_R	QT7F	TTC TTA TGA TTT CAT TCA AAC CC_NED	HpyCH4V
	QT7R	TTG ATT TGA TTA TCA ATC AAT TTC T	

For all markers, restriction enzymes were used (Table 2). The restriction digestion reaction contained 10 μl PCR product, 2.2 μl CutSmart buffer, and 0.6 μl enzyme in a final volume of 20 μl. The reaction lasted 60 min (20 min for QT8F_QT5R) at 37 °C followed by an inactivation at 65 °C (80 °C for QT8F_QT5R) for 20 min. Digestion products were diluted five times before further analyses.

Analyses of all diluted PCR and digestion products were done using an automated ABI Prism Genetic Analyzer (Applied Biosystems) coupled to 3730 Series Data Collection Software 4. Fragment sizes (alleles) were scored using GeneMarker v.2.6.7 (Softgenetics, State College, PA, USA) software.

To control for (cross) contamination, each DNA extraction included two to four negative controls, and each PCR run included one negative control. They were treated in the same way as the normal samples except that no DNA was added. All calculations were done in R 3.6.2^63^.

For both analyses, i.e. determination of the continental origin and of the haplotypes, in case of two successful amplification of the same extract but with different dilutions (1:10 and 1:20), results were compared and as no discrepancies occurred, they were combined.

## Supplementary information

Supplementary Tables.
