# Supplementary material for: DNA of centuries-old timber can reveal its origin
Source: Sci Rep. 2020 Nov 23;10:20316. doi: 10.1038/s41598-020-77387-2 (PMC7683605; doi:10.1038/s41598-020-77387-2)
Supplement: Supplementary file 1 — Supplementary Tables. [file 41598_2020_77387_MOESM1_ESM.pdf]

DNA of centuries-old timber can reveal its origin

Linar Akhmetzyanov, Paul Copini, Ute Sass-Klaassen, Hilke Schroeder, G. Arjen de Groot, Ivo Laros, Aoife Daly

Supporting information

Table S1. PCR products fragment length analyses from historical material, continental provenancing and haplotyping per extraction method

Sample ID	Extraction Method	Continental provenancing markers					Origin	cpSSR markers								HT_cpSSR	RFLP markers			HT_RFLP
		trnCD	psaI-ycf4	psbE-petL	trnDT	trnLF		µdt1	µcd5	µkk4	µdt4	TF-Ivo	µdt3	Cmcs8shrt	µcd4		QT8_QT5	dt72_QT5	QT7	
JMT011_HW	1	0	0	0	0	0		0	0	0	0	0	0	0	0		0	0	0	
	2	120	178	0	0	0	Eu	NA	NA	NA	NA	NA	NA	NA	NA		0	0	100	Not HT7
JMT011_SW	1	0	0	0	86	0	Eu/As	0	0	0	0	0	0	0	0		0	0	0	
	2	0	0	0	0	0		0	0	0	0	0	0	0	0		0	0	0	
JMT031_SW	1	0	0	0	0	0		0	0	0	0	0	0	0	0		0	0	0	
	2	0	0	0	0	0		0	0	0	0	0	0	0	0		0	0	0	
JMT031_HW	1	0	0	0	0	0		0	0	0	0	0	0	0	0		0	0	0	
	2	120	178	0	86	135	Eu	NA	NA	NA	NA	NA	NA	NA	NA		0	0	100	Not H7
ARR031_SW	1	0	0	0	0	0		0	0	0	0	0	0	0	0		0	0	0	
	2	0	0	0	0	0		0	0	0	0	0	0	0	0		0	0	0	
ARR031_HW	1	0	0	0	0	0		0	0	0	0	0	0	0	0		0	0	0	
	2	0	0	0	0	0		NA	NA	NA	NA	NA	NA	NA	NA		28	0	100	H1
SVT031_SW	1	0	0	0	0	0		0	0	0	0	0	0	0	0		0	0	0	
	2	0	0	0	0	0		0	0	0	0	0	0	0	0		0	0	100	Not H7
SVT031_HW	1	0	0	0	0	0		0	0	0	0	0	0	0	0		0	0	0	
	2	0	0	0	0	0		NA	NA	NA	NA	NA	NA	NA	NA		0	0	0	
SVT061_SW	1	0	0	0	0	0		0	0	0	0	0	0	0	0		0	0	0	
	2	0	0	0	0	0		0	0	0	0	0	0	0	0		0	0	0	
SVT061_HW	1	0	0	0	0	0		83	0	0	0	0	0	0	0	10,11	0	0	0	
	2	120	0	0	0	135	Eu/As	NA	NA	NA	NA	NA	NA	NA	NA		0	0	0	
H011001_SW	1	123	0	0	86	0	Eu/As	0	0	0	0	0	0	0	0		91.5	0	0	5,6,7,10,11,12
	2	0	0	0	0	0		0	0	0	0	0	0	0	0		0	0	0	
H011001_HW	1	0	0	0	0	0		83	0	0	0	0	0	0	0	10,11	91.5	0	0	5,6,7,10,11,12
	2	0	0	0	0	0		0	0	0	0	0	0	0	0		0	0	0	

Table S1. Continuation

Sample ID	Extraction Method	Continental provenancing markers					Origin	cpSSR markers								HT_cpSSR	RFLP markers			HT_RFLP
		trnCD	psaI-ycf4	psbE-petL	trnDT	trnLF		µdt1	µcd5	µkk4	µdt4	TF-lvo	µdt3	Cmcs8shrt	µcd4		QT8_QT5	dt72_QT5	QT7	
H011005_HW	1	0	0	0	0	0		0	0	0	0	97	0	58	0		0	0	0	
	2	0	0	0	0	0		0	0	0	0	0	0	0	0		0	0	0	
F042003_SW	1	0	0	0	86	0	Eu/As	0	0	0	0	84	0	0	0		0	0	0	
	2	0	0	0	0	0		0	0	0	0	0	0	0	0		0	0	0	
F042003_HW	1	0	0	0	0	0		0	0	111	0	84	0	58	0	1	0	0	0	
	2	0	0	0	0	0		0	0	0	0	0	0	0	0		0	0	0	
F042010_SW	1	0	0	0	0	0		0	0	0	0	84	0	0	0		0	203	0	4,5,6,7
	2	0	178	185	86	0	Eu	82	75	0	0	84	0	58	96	1,7	0	0	0	
F042010_HW	1	0	0	0	86/101	135	Eu/As	82	75	110	0	0	126	0	0	7	91.5	203	174	7
	2	0	0	0	0	0		0	0	0	0	0	0	0	0		0	0	0	
F042011_HW	1	0	0	0	0	0		82	75	0	0	0	0	0	0	1,7	0	0	0	
	2	0	0	0	0	0		0	0	0	0	0	0	0	0		0	0	0	
F042011_SW	2	0	0	0	0	135	Eu/As	82	75	0	0	84	0	0	0	1,7	0	0	100	Not H7
JK-I_HW	1	0	0	0	0	135	Eu/As	0	0	0	143	0	0	0	0	10,11,12	0	0	0	
	2	0	178	0	0		Eu/N Am	0	0	0	0	0	0	0	0		0	0	0	
JK-B_HW	1	0	0	0	0	0		0	0	110	0	0	0	0	0	7,10,11,12	0	0	0	
	2	0	0	0	0	0		0	0	0	0	0	0	0	0		0	0	0	
JKd2_SW	1	0	178	185	0	0	Eu/N Am	83	0	0	0	84	0	58	0	12	0	0	0	
	2	0	0	0	0	0		NA	NA	NA	NA	NA	NA	0	0		0	0	0	
JKd2_HW	1	0	0	0	0	0		0	0	0	0	0	0	0	0		0	0	0	
	2	0	0	0	0	0		0	0	0	0	0	0	0	0		0	0	0	
CesDt2_SW	1	123	0	0	86	0	Eu/As	82	0	0	0	84	0	0	0	1,7	0	0	0	
	2	0	0	0	0	0		NA	NA	NA	NA	NA	NA	0	0		0	0	0	

Table S1. Continuation

Sample ID	Extraction Method	Continental provenancing markers					Origin	cpSSR markers								HT_cpSSR	RFLP markers			HT_RFLP
		trnCD	psaI-ycf4	psbE-petL	trnDT	trnLF		µdt1	µcd5	µkk4	µdt4	TF-Ivo	µdt3	Cmcs8shrt	µcd4		QT8_QT5	dt72_QT5	QT7	
CesDt2_HW	1	0	0	0	0	0		0	0	0	0	0	0	0	0		0	0	0	
	2	0	0	0	0	0		NA	NA	NA	NA	NA	NA	NA	NA		0	0	0	
CesDt3_SW	1	0	0	0	86	0	Eu/As	82	0	0	0	84	0	0	0	1,7	0	0	0	
	2	120	0	0	0	0	Eu/As	NA	NA	NA	NA	NA	NA	NA	NA		0	0	0	
CesDt3_HW	1	0	0	0	0	0		0	0	0	0	0	0	0	0		0	0	0	
CesDt5_HW	1	0	0	0	0	0		0	0	0	0	0	0	0	0		0	0	0	
	2	0	0	0	0	0		NA	NA	NA	NA	NA	NA	NA	NA		0	0	0	
CRL32__SW	1	0	0	0	0	0		0	0	0	0	0	0	0	0		0	0	0	
	2	0	0	0	0	0		NA	NA	NA	NA	NA	NA	NA	NA		0	0	0	
CRL32__HW	1	0	0	0	0	0		0	0	0	0	0	0	0	0		0	0	0	
	2	0	0	0	0	0		NA	NA	NA	NA	NA	NA	0	0		0	0	0	
Z255002_SW	1	123	178	185	86	135	Eu	83	76	110	0	84	125	58	96	12	NA	NA	NA	
Z255002_HW	1	123	178	185	86	135	Eu	83	76	110	0	84	125	58	96	12	NA	NA	NA	
Z018.605.029_HW	1	0	0	0	0	0		0	0	0	0	0	0	0	0		NA	NA	NA	
Z018.605.040_HW	1	0	0	0	0	0		0	0	0	0	0	0	0	0		NA	NA	NA	
Z092319a_HW	1	0	0	0	0	0		0	0	0	0	0	0	0	0		NA	NA	NA	
Z092319a_SW	1	0	0	0	0	0		0	0	0	0	0	0	0	0		NA	NA	NA	
Z092313a_HW	1	0	0	0	0	0		0	0	0	0	0	0	0	0		NA	NA	NA	
Z092314a_HW	1	0	0	0	0	0		0	0	0	0	0	0	0	0		NA	NA	NA	
Z092324b_HW	1	0	0	0	0	0		0	0	0	0	0	0	0	0		NA	NA	NA	
Z223004_SW	1	0	0	0	0	0		0	0	0	0	0	0	0	0		NA	NA	NA	
Z223004_HW	1	0	0	0	0	135	Eu/As	84	0	0	0	84	0	58	0	10	NA	NA	NA	

1, 2 - extraction Protocol 1 and extraction Protocol 2, respectively
Origin – identified continental origin of the sample based on continental provenancing analyses
CP, cpSSR and RFLP – continental provenancing, chloroplast simple sequence repeat and restricted fragment length analyses, respectively
HT_cpSSR and HT_RFLP –sample haplotype based on cpSSR analyses and sample haplotype based on RFLP analyses, respectively
Eu, As and NAm – Europe, Asia and North America, respectively
HW, SW – heartwood and sapwood, respectively. Given in column “Sample ID” .

Table S2. Primers sequence designed for discrimination between haplotypes 10 and 11

Primer	Sequence 5‘ -3‘
TF-Ivo_F	PET_TGACGACCCGAATCTTTATTTATTT
TF-Ivo_R_PIG	GTTTCTTGGATCGATTCACAACAACTCTTTC

Table S3. PCR products fragment length analyses for continental provenancing of material from living oak trees

**Sample ID**	**Marker**				
	**trnDT**	**psbE-petL**	**psaI-ycf4**	**trnLF**	**trnCD**
JUG020H	0	0	0	0	0
JUG008H	86	185	178	135	123
GOR005H	86	185	178	135	123
GOR007H	86	185	178	135	123
JUG009H	86	0	178	135	123
SVA004H	86	185	178	135	123
OTE002H	86	185	178	135	123
SVA002H	0	0	0	0	0
OTE001H	86	185	178	135	123
SVA003H	86	185	178	135	123
JUG016H	0	0	0	0	0
JUG004H	86	185	178	135	123
JUG006H	0	0	0	0	0
JUG023H	86	185	178	135	123
JUG007H	86	185	178	135	123
GOR022H	0	0	0	0	0
JUG024H	0	0	0	0	0
GOR006H	0	0	0	0	0
JUG020S	86	185	178	135	123
JUG008S	86	185	178	135	123
GOR005S	86	185	178	135	123
GOR007S	0	0	0	0	0
JUG009S	86	185	178	135	123
SVA004S	86	185	178	135	123
OTE002	86	185	178	135	123
SVA002	86	185	178	135	123
OTE001	86	185	178	135	123
SVA003	86	185	178	135	123
JUG016	86	185	178	135	123
JUG004	0	0	0	0	0
JUG006	86	185	178	135	123
JUG023	86	185	178	135	123
JUG007	86	185	178	135	123
GOR022	0	0	0	0	0
JUG024	86	185	178	135	123
GOR006	86	185	178	135	123
